# Perspectives of paddy workers regarding the use of sunscreen: a theory-based qualitative research

**DOI:** 10.1186/s13690-019-0361-1

**Published:** 2019-08-01

**Authors:** Hadiseh Panahi, Leili Salehi

**Affiliations:** 1 Health Education & Promotion Department, Alborz University of Medical Sciences, Karaj, Iran; 2 Health Education & Promotion Department, Research Center for Health, Safety and Environment, Alborz University of Medical Sciences, Karaj, Iran

**Keywords:** Skin cancer, Sunscreen, Health action process approach, Qualitative study

## Abstract

**Background:**

Skin cancer is one of the most frequent types of cancer. This study aims to clarify farmers’ perspectives regarding the use of sunscreen according to Health Action Process Approach.

**Method:**

Twenty-seven farmers were recruited an interviewed. The samples were classified according to their age, sex, and education. The data were collected through a deep and semi-structure interview during one-month period. Questions were asked based on Health Action Process Approach (HAPA) and were continued until the saturation stage.

**Results:**

The data were classified into six categories (intention, risk perception, outcome expectation, self-efficacy, perceived barriers and action planning). Most of the interviewees did not use sunscreen and did not intend to use it either. They indicated some threats of sunlight, such as burn and rash, redness, itching, soreness and darkness of skin. The participants pointed to some physical outcome expectation of the sunscreen and mentioned some perceived barriers such as time limitation, low income, non-familiarity with sunscreen, the notion that sunscreen is only for women and the farmers prioritize farming and believed that sunscreen is not required in all seasons.

**Conclusions:**

Risk perception, outcome expectation and perceived barriers should be considered designing appropriate interventions. The education of protection behaviors should be considering during interventional strategies.

## Background

Currently skin cancer is considered one of the most frequent types of cancers [1]. The annual frequency of malign dermal in the US is about 3% [2]. The treatment is expensive and the spread of the disease causes a lot of financial difficulties for families [3]. In Iran, skin cancer is the most common cancer, occurring at a frequency of 10 or 13 cases per 100,000 people [4].

Most of these cases involve farmers [5]. Sunlight is one of the most important factors in skin cancer affliction [6]. In addition to skin cancer, sunlight causes early aging, photo dermatitis, and active keratosis [7]. According to a study conducted by Hommanal et al., construction workers, gardeners, and municipality workers are among those with a high risk of skin cancer [8]. Due to a systematic study in 2016, sunlight is one of the risk factors in work. Those who work outdoors are exposed to ultraviolet rays and its dangers, such as skin cancer [9].

Skin protection mechanisms include using sunscreen and wearing suitable clothes (outfit). Using sunscreen helps people enjoy the benefits of sunshine while doing outdoor activities [10] and is more beneficial than other methods. Considering the available instruction and strategies, using sunscreen is effective if it is used every 2 hours [11]. The current evidence shows that 29 to 50% of people do not make use of proper mechanisms against sunshine [12]. For instance, according to a study 48% of farmers have never used sunscreen while they are exposed to sunshine more than others. Thus implementation of appropriate interventions to encourage people including farmers to use sunscreen is of prime importance. However, as recommended these interventions should be theory driven in order to be effective and appealing to the target audience. One such theory is Health Action Process Approach (HAPA). The HAPA is consisted of two phases (motivational& volitional). In the motivational phase, a person plans to rectify his/her behavior, which may lead to risk. In the motivational phase, risk perception, outcome expectancies, and task self-efficacy play an essential role. In the intentional phase, action planning, coping planning, and coping self-efficacy are the key points [13].

Since little information was available on the use of sunscreen by Iranian paddy workers, and since no study was previously conducted on the experiences of paddy workers about sunscreen use, this qualitative study aimed to explore the topic. It was hoped that the findings from this study could help to provide appropriate intervention for sunscreen use by farmers. This study aims to clarify farmers’ perspectives regarding the use of sunscreen according to Health Action Process Approach.

## Methods

### Study design

This was a qualitative study, using the content analysis approach to investigate farmers’ perspectives on using sunscreen based on the HAPA.

### Recruitment

The sample for the study included paddy workers and were recruited from the northern part of the Iran (Gilan Province). Inclusion criteria was: age equal or above 30 years old, fluent in speaking Persian and willing to participate in the study. The farmers who worked less than 2 hours a day on the farms and had less than 5 years experiences were excluded.

### Sampling strategy

A stratified sampling method was used to select villages (four villages out of 460). The list of villages was obtained from Rudsar agricultural authorities. First, within each region (north, south, east and west), a village was selected. Then within each village a purposive sample of farmers with the consideration of maximum variation in terms of age, wok experiences and education was entered into the study. If the farmer had the inclusion criteria and willing to participate in the study, the interview was carried out, and if not, another farmer was considered from the same agricultural land or an adjacent agricultural land. In all instances the setting was the farmers’ natural working place.

### Data collection

Face to face interviews with interview guide were conducted to collect the data. The interview questions were formulated based on the HAPA and were continued until saturation [14]. In fact, during the process of study design research team provided a purpose statement, and then they listed the questions related to key theme associated with the HAPA model. During the interviews explorative questions about the predetermined codes were asked. Sample items included ‘What do you know about sun burning?’ What did you do for preventing yourself from sun burning?’ please explain your experiences about sun burning and sunscreen use?’ why are you did not using sunscreen? ‘Please explain about benefits of sunscreen use’ and ‘please tell me are you going to use sunscreen when you are working on land’. The length of the interview was about 20–30 min and the participants were asked about their individual characteristics (age, sex, education, farming experience, daily working hours, and sunburn history) and the questions regarding the HAPA constructs. All the interviews were recoded by voice recorder and taped immediately after the session of each interview.

Then the researcher went to farmers’ workplaces on agricultural land and introduced her-self and interviewed the farmers. The interviewer used ones’ highest communicative capability to make a relationship with farmers. The participants were informed about the aim and length of the interview and that their privacy would be maintained. The first author (HP- MS student with experiences of conducting interview) performed interviews. The interviewer attempted to remain as neutral as possible and encouraged responses.

### Data analysis

Directed content analysis was used for data analysis. We used inductive approach due to limited previous finding in this regards. This method of qualitative study is one of a variety of qualitative analysis methods that its initial code begins with regard to the theory. Its purpose is to develop a conceptual framework or previous theory. The theory chosen in this type of study can be about the prediction of the desired variable and the relationships between variables. As a result, the code is useful in how to initialize the coding. The definitions of each category are determined using the desired theory [15]. The data of the study were transcribed by the researchers and based on HPAP. Both type of the manifest and latent content were considered for content analysis [16]. We identified the predetermined codes, then we began coding immediately and all the data which not matched to any code were analyzed to determine if they represent a new category or subcategory of an existence codes. The codes were then classified into sex categories: intention, risk perception, outcome expectancies, self-efficacy, and perceived barriers and action planning. Interview transcriptions were considered as the meaning unit.

### Rigor

In order to evaluate the credibility of the data, prolonged engagement and member check was used. In order to verify the code placement in categories, external check was carried out (experts’ panel). Comparing codes few days after the first coding, recoding was used for data reliability. The obtained data were then presented to the participants for confirming credibility based on their feedback. The dependability of the study was analyzed through external check by another researcher. For conformability, the opposite comments were investigated to identifying the reasons.

### Ethics

The ethics committee of Alborz University of Medical Sciences approved the study (Ethical Code: IR.ABZUMS.REC.1397.064). For audio taping interview content, the participants’ permission was obtained. All the participants were informed about the purpose of the study and if any participant was not willing to participate in study, he/she was excluded written consent form was signed by each participant.

## Results

### Participants

In all 27 paddy farmers (12 male and 15 female) were interviewed during one-month period (from 11 July to 11 August 2018). The mean (SD) age of participants was 57.46 (15.75) years. Their average working hour was 7 hours a day (ranging from four to eight). The characteristics of the participants are shown in Table 1.

**Table 1:** Demographic traits of interviews

Variable	Number	Percent
Age		
30–45	9	33.33
46–60	11	40.74
61–75	7	25.93
Gender		
Woman	15	55.56
Man	12	44.44
Farming Experience		
15–30	15	55.56
30–45	7	25.92
45–60	5	18.52
Sunburn History		
Yes	27	100
No	0	0
Education		
Illiterate	5	18.52
Literate	22	81.48
Farming Experiences		
5–15	14	51.85
15–30	13	48.15

All the interviewees stated that they were suffering from sunburn, redness of skin, darkness, rash, irritation and itching of the skins and about 92% of them had never taken any action in sunburn prevention and just used cold water to protect their head and facial skin against sunburn. Only two participants indicated that they were using sunscreen as received advice from their doctors and daughters. They were using sunscreen for 3 and 6 years, respectively, and were satisfied with the results.

### Categories

Regarding the use of sunscreens, 27 codes were extracted and classified into six categories of predetermined HAPA (intention, risk perception, outcome expectancies, perceived barriers, and self-efficacy and action planning) (Table 2).

**Table 2:** Meaning unit, codes and categories

Meaning Unit	Summarized meaning Unit	Code	Categories
“I do not intend to use a sunscreen. I think using a wicker hat and long sleeve clothing will protect me well from sunlight.”	No intention to use sunscreen due to use other protectors	The tendency to use sun screen	Intention
“I do not intend to use it, no one has ever taught us about the benefits of the cream. In this case, we may be able to use it in a proper way”	No intention to use sunscreen due to lake of knowledge		
“In the farming season, we are all busy and no one thinks about using sunscreen. Who has the time?”	Not using sunscreen due to lack of time	Time limitation	Perceived barriers
“I know that not using sunscreen causes sunburn and rash, but I don’t have enough time to use it.”			
“I have never used sunscreen and I don’t think it is necessary for men to use it.”	Not using sunscreen due to gender	Gender limitation	
“I have never used sunscreen because I think it is a kind of cosmetic.”	Not using sunscreen due to cosmetic nature	Cosmic limitation	
“I have never used sunscreen. I use a straw hat instead and I think it has the same effect.”	Not using sunscreen due to using another protector	Using another shelter	
“Being exposed to sunshine causes sunburn.”	Sunshine dangers	Hazards	Risk perception
“Being exposed to sunshine causes rash and skin darkening.”	Sunshine dangers		
“I think, covering the skin against sunshine can be a prevent from skin cancer and I do not need additional planning method to use sunscreen”		Planning	Action planning

#### (i) The intention of using the sunscreen

According to the findings of this study, the majority of the farmers who were interviewed did not intend to use sunscreen. Moreover, they thought that it is health centers and agricultural ministry tasks to teach them about use of sunscreen.

“No one has ever taught us about the benefits of the sunscreen. In this case, we may be able to use it in a proper way.” (*Woman, 56 years old, literate*).

“No one has taught us and, in such cases, agriculture department must teach farmers individually. If some people come and talk about its benefits, we can trust him/her and use it.” (*Man, 60 years old, literate*)

#### (ii) barrier to the use of sunscreen

Almost none of the participants (92%) used sunscreen. Due to the obstacles related to usage of sunscreen in this study, the main barriers are mentioned here: Time and income limitation, not having enough information about its function, gender limitation, priority of farming, not needing sunscreen in any of the seasons, the adequacy of using a straw hat, long-sleeved shirts, gloves, and scarves. Moreover, the farmers are required to travel a long distance from their houses in the village to the city in order to buy the sunscreen. Furthermore, they are not interested in using sunscreen and consider it as a cosmetic. Almost none of them were aware of the SPF (Sun Protection Factor) function and its application in suitable amounts.

“If the government offers it at a reasonable price or if our income increases, we may use it” (*Man, 70 years old, illiterate*)

“I have been using sunscreen for six years. Due to skin redness and itching. I went to the doctor and he prescribed some medicine and sunscreen. I’m satisfied with its effectiveness on my skin. I need it on sunny days and during work there is no need to use it on cloudy days.” (*Woman, 58 years old, literate*)

#### (iii). Risk perception

In this study, all the cases were subject to sunburn. All but one was aware of dangers associated with sunburn, which occurs due to working for long hours under the sun. The participants named some threats such as sunburn and rash, skin darkness, and redness. Two of them mentioned skin cancer but they believed that a straw hat and a proper outfit can protect them against it. Headache and sore eyes were the other problems that were mentioned while working for a long time under the sun.

#### (iv) Self-efficacy

Almost all the participants believed that if they fully realized that sunscreen is good for them, they would use it.

“If health personal conduct some classes and explain the advantages of the sunscreen, we can use it.” (*Man, 50 years old, literate*)

“No one has ever taught us in this regard.” (*Man, 50 years old, illiterate*)

#### (v) Outcome expectancies

Most of the interviewees in this study were not aware of the benefits of sunscreen. Two of them believed that sunshine can be beneficial for the skin and there was no need to use sunscreen. Some believed that not using sunscreen is harmful but 99% of them did not know the preventive procedures against skin cancer.

“Not using sunscreen can lead to rash and scald.” (*Woman, 60 years old, literate*)

“Definitely, not using the sunscreen can have some side effects and harm the skin.” (*Man, 30 years old, literate*)

“Sunscreen can be useful. I saw sunscreen advertising on TV.” (*Woman, 60 years old, literate*)

#### (vi). Action planning

Participants in current study were not having any planning to use sunscreen. They believed that covering the skin against sunrays can be a preventive method against skin cancer. Most of the participants received their information via personal experiences or talking with others. Some had heard about the benefits of sunscreen from TV or radio and believed that health clinics and agriculture department must offer the necessary education to them.

## Discussion

This qualitative study was done in the northern part of the Iran to clarify paddy workers’ perspectives on using sunscreen according to the HAPA. Based on the current study findings, the majorities of the participants neither used sunscreen nor intended to use it. A study in this field was done on Australian farmers, 54% were never use sunscreen [17]. According to a study by Fishbin and Ajzen in 1975, intention is a key parameter in changing behavior. Some researchers believed that there is no permanent relation between intention and behavior and there is a gap between them [18]. In this regard, Jones study has shown that intention consists of just 44% variance in using sunscreen [19].

According to this study results almost all the interviewees were aware of sunlight’s possible threat. According to a study conducted by Tabatabaiyan et al. [20] 53% of people were aware of the harmful effects of sun rays. This is lower than the percentage found in the current study. The current study was conducted on farmers who had been working for more than 5 years but Tabatabaiyan study was conducted on students, who were not exposed to sun rays as much as the farmers. Therefore, this difference seems reasonable.

In the current study, just 3.7% of the participants mentioned the dangers of sun rays. Furthermore; participants believed the risk of skin cancer could be eliminated by straw hat and long sleeved shirt. In a study conducted by Kim et al. (2009), this was about 65% [21]. Since Kim et al. conducted the study in a skin clinic, this difference is quite reasonable. In another study done by Pichon et al., 76% of people either did not consider themselves as likely to get skin cancer [22]. Those who considered themselves at risk of skin cancer either had a record of skin cancer in the family or had sensitive skin. In a study conducted by Mazloomi et al., 12% of the teachers were not aware about sunscreen roles against skin cancer [23]. There is a positive significant relationship between risk perception and using sunscreen [24] if a person is not aware of the his/her behavior results,it is impossible to change behavior [25].

Due to this study finding almost all the people being interviewed would have used the sunscreen if they knew that it was good for them. In other studies, self-efficacy leads to a change in behavior [26] and increases the use of sunscreens [27].

Based on the current study results, physical outcome expectancies was emphasized than other levels by interviewees sunscreen protect skin from sunburn, itching, scalding, redness and so on. Almost all the participants in the current study would use the sunscreen if they become aware of its usefulness.

Various perceived barriers are mentioned in this study, such as time limitation, low income, not being familiar with sunscreen and its function, gender limitation, priority of working, not needing it in different seasons, adequacy of using a straw hat and gloves, wearing long-sleeved shirts and scarves and so on. Inability to buy sunscreen as the farmers stayed away from the city, disinterest in using sunscreen, and considering it to be a cosmetic was the most important factors of not using sunscreen. In other research studies, some factors such as the costs, inaccessibility, inconvenience, and cultural differences are mentioned as an obstacle in using sunscreen [28]. In a study in Turkey, it was shown that the percentage of men who have never used sunscreen exceeds that of the women [28]. It is obvious that by using some time management strategies and financial planning, one can overcome the problems. By consulting with local shopkeepers and justifying them to order sunscreen with a suitable SPF, farmers would have an easier access to sunscreen.

This study had some strengths and limitations. Two major strengths were the fact that we used a theoretical framework (HAPA) for the study; and the fact that to the best of our knowledge, this is the first qualitative study that explores Iranian farmers’ views on using sunscreen. However, there were several limitations for the study. First, since the study was carried out in agricultural season, some factors such as lack of time might be influenced data collection process, although we tried to adjust the interview time accordance with participant’s situations. Second Given that this study was conducted among Iranians north farmers, the findings of this study might not be generalized to all Iranian’s farmers. These farmers might differ from others in terms of socioeconomic status, age, skin sensitivity.

## Conclusions

In total, according to the findings of this study, in order to improve sunscreen use among Iranians farmers, perceived barriers, outcome expectation and risk perception should receive more attention. Indeed, it is essential to plan health education programs and protection interventions for this group of the population. Of course, attention to the appropriate channels of communication is essential.

## Data Availability

All datasets in this study are available from the corresponding author in request.

## Abbreviation

HAPA: Health Action Process Approach
